# Controversies about the enhanced vulnerability of the adolescent brain to develop addiction

**DOI:** 10.3389/fphar.2013.00118

**Published:** 2013-11-28

**Authors:** Aurélien Bernheim, Olivier Halfon, Benjamin Boutrel

**Affiliations:** ^1^Center for Psychiatric Neuroscience, Division of Child and Adolescent Psychiatry, Lausanne University HospitalLausanne, Switzerland; ^2^Division of Child and Adolescent Psychiatry, Lausanne University HospitalLausanne, Switzerland

**Keywords:** drug addiction, adolescence, impulsivity, brain imaging, animal models

## Abstract

Adolescence, defined as a transition phase toward autonomy and independence, is a natural time of learning and adjustment, particularly in the setting of long-term goals and personal aspirations. It also is a period of heightened sensation seeking, including risk taking and reckless behaviors, which is a major cause of morbidity and mortality among teenagers. Recent observations suggest that a relative immaturity in frontal cortical neural systems may underlie the adolescent propensity for uninhibited risk taking and hazardous behaviors. However, converging preclinical and clinical studies do not support a simple model of frontal cortical immaturity, and there is substantial evidence that adolescents engage in dangerous activities, including drug abuse, despite knowing and understanding the risks involved. Therefore, a current consensus considers that much brain development during adolescence occurs in brain regions and systems that are critically involved in the perception and evaluation of risk and reward, leading to important changes in social and affective processing. Hence, rather than naive, immature and vulnerable, the adolescent brain, particularly the prefrontal cortex, should be considered as prewired for expecting novel experiences. In this perspective, thrill seeking may not represent a danger but rather a window of opportunities permitting the development of cognitive control through multiple experiences. However, if the maturation of brain systems implicated in self-regulation is contextually dependent, it is important to understand which experiences matter most. In particular, it is essential to unveil the underpinning mechanisms by which recurrent adverse episodes of stress or unrestricted access to drugs can shape the adolescent brain and potentially trigger life-long maladaptive responses.

## INTRODUCTION

A common consideration on addiction disorders acknowledges that individual characteristics may predispose to drug addiction; meanwhile excessive drug intake still is considered to influence personal traits and promote compulsive drug consumption (Swendsen and Le Moal, 2011). The vast majority of drug users are teenagers and young adults or began consuming during adolescence (O’Loughlin et al., 2009). In particular, a recent report of the National Survey on Drug Use and Health indicated that 31.2% of people below the age of 25 had consumed illicit drugs during the past month, while only 6.3% of older people acknowledged to do so (Substance Abuse and Mental Health Services Administration, 2010). The younger teenagers start using drugs, the more severe signs of drug addiction are. Among people in the USA that tried marijuana before the age of 14, 12.6% developed signs of drug abuse or dependence, while only 2.1% of those experiencing marijuana after the age of 18 suffered from severe signs of dependence (Substance Abuse and Mental Health Services Administration, 2010).

Adolescent risk-taking and reckless behavior is a major public health concern that increases the odds of poor lifetime outcomes, including loss of control over drug use. Compelling evidence based on imaging technologies have shown that brain circuitries involved in affective and cognitive processes interact dynamically across development. At the cellular level, these changes correspond with the marked overproduction of axons and synapses in early puberty, and rapid pruning in later adolescence and young adulthood. The current consensus considers that patterns of neural connection among systems of emotion, motivation and cognitive processes related to the pursuit of long-term goals undergo a natural reorganization and a set of maturational refinements during adolescence (Gogtay et al., 2004; Giedd, 2008). In contrast to the relatively early and rapid changes in affective systems that appear to be linked to pubertal maturation, another set of cognitive skills and competence in self-control seem to develop gradually across adolescence and continue to mature long after puberty is over (Dahl, 2008). This key observation may explain why adolescence is characterized by an imbalance between the relative influences of motivational and control systems on behavior (Somerville et al., 2011). As a consequence, the adolescent brain is a tempted brain as long as the development of executive functions including relevant decision making and planning, abstract reasoning and response inhibition remains unfinished (Dahl, 2008).

In this perspective, taking drugs during adolescence may interfere with the normal brain development, and may increase the vulnerability to abuse drugs later during adulthood (Andersen, 2003; Crews et al., 2007). Despite the growing number of prevention campaigns, drug consumption in adolescents remains quite stable over the past years. Strikingly, a relevant communication released in 1952 already acknowledged that “*drug addiction in adolescence is not a new phenomenon*” (Zimmering et al., 1952), and the ultimate question was already clearly identified “*However, there is still the question of why, under apparently similar external conditions, some boys will try the drugs and others won’t, why some go down the road of addiction while others give up the drug* (…).” Sixty years later, this question remains partially unanswered. Animal models, especially rodents, have contributed to a better comprehension of the juvenile state. In particular, converging evidence has pointed out to an enhanced vulnerability to drug abuse in adolescents, but questions and controversies remain regarding the relevance of the different animal models and the interpretation of the data (Schramm-Sapyta et al., 2009). Interestingly, these authors conclude that even if an increased recreational drug use is usually observed during adolescence, evidence relating to pathological drug seeking and taking still is lacking. In this review, we try to summarize the biological factors relevant to adolescent driving risks and we discuss the clinical observations in the light of preclinical findings linking impulsivity and emotional reactivity to initiation of drug use and risks of abuse.

## PUBERTY AND ADOLESCENCE

Risk taking during adolescence is the product of an interaction between heightened stimulation seeking and an immature self-regulatory system that is not yet able to modulate reward-seeking impulses (Steinberg and Morris, 2001; Steinberg, 2004, 2005). A consensus could put adolescents at risk for emotional and behavioral disorders. Nevertheless, increased risk and novelty seeking can be beneficial for learning novel strategies for survival (Kelley et al., 2004). Indeed, from an anthropologic perspective, some types of risk taking can be viewed as an adaptive willingness to demonstrate bravery in order to acquire a better social status. In many situations, it seems that adolescent do not become more fearless after puberty but rather they may become more highly motivated to act boldly despite their fears, particularly when they perceive that acting in a brave or reckless way might bring them increased recognition by peers (Dahl, 2008).

The period of adolescence is a time of considerable change, as sex-specific pubertal hormones bring about changes in physical stature, reproductive organs and other secondary sexual characteristics. Neuroendocrine changes during puberty influence behavioral and emotional development (Waylen and Wolke, 2004). Since testosterone cross the blood brain barrier (Pardridge and Mietus, 1979), it contributes to the cortical pruning during adolescence, especially in frontal and temporal lobes (Witte et al., 2010; Nguyen et al., 2013). This observation is of interest and may explain sexual dimorphism in gray matter and its behavioral consequences (Neufang et al., 2009; Paus et al., 2010; Bramen et al., 2012).

A classical strategy to assess this influence is to select adolescents of similar age, but experiencing different stage of puberty. Mid-late puberty adolescents differ from adolescents in early puberty in their emotional regulation of startle response and postauricular reflex, two physiological measure of defensive and appetitive motivation (Quevedo et al., 2009). Similar results have been reported with mid/late puberty adolescents displaying an enhanced pupil dilatation in response to emotional words (Silk et al., 2009).

## GRADUAL EMERGENCE OF COGNITIVE SELF-CONTROL DURING ADOLESCENCE: INSIGHT FROM NEUROIMAGING

The adolescent behavior, marked by intense affective expression and impulsive responses, has long been studied, but the most recent imaging technologies have contributed to a better knowledge of the developing brain during adolescence. In particular, it has been shown that proportion of gray matter decreases whereas white matter increases during transition from childhood to young adulthood (Paus et al., 1999; Lenroot and Giedd, 2006). Whereas the enhanced myelination follows a quite linear pattern all over the brain, with only slight local variations, the diminution of gray matter, also called synaptic pruning, is more selective. Hence, myelination is not only considered as an electrical insulator that increases the speed of neuronal signal transmission, but also as a key process that modulates the timing and synchrony of neuronal firing patterns that convey meaning in the brain (Giedd, 2008). The main neurobiological changes that account for risky behaviors in adolescence occur in the mesocorticolimbic system, particularly in the prefrontal structures (Chambers et al., 2003; Crews et al., 2007; Crews and Boettiger, 2009). Studies comparing adult and adolescent cortical function indicate that adolescent process information differently, often enlisting different brain regions than adults. Difficulty with executive cognitive functioning and behavioral self-control, including difficulties with planning, attention, foresight, abstract reasoning, judgment, and self-monitoring have been reported in adolescents, and several functional magnetic resonance imaging (fMRI) studies have examined the functional neuroanatomy underlying executive processing in children, adolescent and adults (Luna et al., 2010). This growing body of evidence supports the idea that frontostriatal systems undergo significant remodeling in the period from adolescence to young adulthood. Specifically, protracted development of prefrontal cortex (PFC), in concert with an amplified motivational drive mediated by the striatum, is thought to be critical to increased novelty seeking and suboptimal decision making that leads to risky behavior and experimental drug use. Assuming that orbitofrontal cortex (OFC) is critical to making value decisions, individual differences in the development of this region might increase or decrease sensitivity to reward through suboptimal computation of incentive value based on reward magnitude coded by the striatum. Conversely, reduced orbitofrontal modulation of striatal-mediated motivational drive could lead to increased novelty seeking and impulsive choice. In either case, significant imbalance in the neurodevelopmental trajectory of this circuit could lead to loss of self-control during a vulnerable period (Yurgelun-Todd, 2007).

The immature connections between the PFC, the nucleus accumbens (Nacc) and the amygdala have been proposed to largely influence goal-directed behaviors in adolescents (Galvan et al., 2006; Ernst et al., 2009). In particular, it has been shown that teenagers engage the orbitofrontal cortex to a much lesser extent compared to adults when facing risky choices. Similarly, adolescents have been also shown to display a decreased and uncoordinated neuronal processing in the OFC during simple reward-related behavior (Sturman and Moghaddam, 2011). These types of observation may partially explain the increased propensity for reckless behaviors during adolescence (Eshel et al., 2007). Finally, in order to emphasize the adolescent brain immaturity upon reward expectations, compelling evidence recently demonstrated a linear reduction of insular activation along with age, with early adolescents displaying the higher activation and late adolescents exhibiting the most reduced signal while gambling in a slot machine task (Van Leijenhorst et al., 2010).

Several epidemiological researches support the idea that adolescence is the life period with the highest rate of impulsive behavior (Steinberg et al., 2008; Romer et al., 2009). Steinberg and colleagues described a linear decrease of impulsivity from the age of 10–30: using different age cohorts, steeper delay discounting and weaker performances on the IOWA gambling task (IGT) have been reported in adolescents, compared to adults (Steinberg et al., 2009; Cauffman et al., 2010). A longitudinal study using the IGT in adolescents aged from 11 to 18 confirmed this result by showing that performance improved continuously with age (Overman et al., 2004). These observations are thought to mirror the maturation of the PFC, which allows the transition from impulsive to more controlled choices. Conversely, an inverted-U shape curve for sensation seeking has been reported as well, with a peak around age 14 (Steinberg et al., 2008). Again, the dissociation between the progressive development of impulse control and the non-linear development of the reward system may result in a misbalance that enhances impulsive choices for reward (Ernst et al., 2009).

Converging fMRI studies exploring decision-making tasks have shown that adolescents and adults share many similarities in neurocircuitry activation, but they also display intriguing differences. A greater response in the left Nacc was reported in teenagers while adults displayed an increased activation in the left amygdala (Ernst et al., 2005). Galvan et al. (2006) also reported enhanced Nacc response to reward in adolescent compared to adults, as well as reduced activation in areas of the frontal cortex. Most recently, in a study examining risk taking in monetary decision-making, it has been shown that adolescents displayed a reduced activation in regions of the OFC compared with adults, and reduced activity in these frontal brain regions was correlated with greater risk-taking tendencies in teens (Eshel et al., 2007). These findings suggest that adolescents engage relatively fewer prefrontal regulatory processes than adults when making decisions. Consequently teenagers may be more prone to risk taking in certain situations. In other words, reduced prefrontal cognitive control may authorize a greater influence of affective systems that dictate decision making and behavior which, in turn, increases adolescent vulnerability to social and peer contexts that activate strong feelings (Dahl, 2008).

In a recent study aiming at assessing adolescent and adult behaviors in a video driving game, it has been shown that adolescent participants took more risks, focused more on the benefits than the costs of risky behavior, and made riskier decisions when surrounded by peers compared to adults (Gardner and Steinberg, 2005). These findings confirm that adolescents may be more prone to peer influences on risky decision-making, and that peer influence (and other social-context variables) may play an important role in explaining reckless behaviors during adolescence. Interestingly, it has been established that young adolescents, categorized as highly resistant to peer influence, displayed enhanced brain connectivity, especially in the frontal cortex, compared to adolescents categorized as highly influenced by peers (Grosbras et al., 2007). Resistance to peer influence has also been positively correlated with ventral striatum activation, but negatively correlated with activation in the amygdala (Pfeifer et al., 2011). Specific pattern of cortical activation in adolescents has been reported by using mentalizing, face recognition and theory of mind tasks. For example, early adolescents aged from 10 to 14 engaged more their medial PFC than adults to analyze the intent of a drawing (sincere or ironic), despite similar performance on the task (Wang et al., 2006). This might reflect a greater effort for the youngsters to perceive social emotional situations they are not yet used to, while adults analyze these situations more effectively, based upon previous experiences.

Noteworthy, adolescence also represents a particular period of emotional perception and regulation. Cognition and decision-making processes in adolescents are highly influenced by their emotional state, a phenomenon called hot cognition (in opposition to cool cognition, in which decision-making occurs under low emotional level). Adolescents also seem to be more sensitive to stressful stimuli. The rate of cortisol release after a stressful task displayed a linear increase with age, in young adolescents aged from 9 to 15 years (Gunnar et al., 2009; Stroud et al., 2009). Presenting fearful faces, induced a higher reactivity of the amygdala in adolescents compared with children and adults (Hare et al., 2008). Interestingly, the habituation of amygdala activity to these fearful faces was lower in subjects screened for high trait anxiety. This enhanced sensitivity to stressful stimuli, together with a higher proportion of hot cognition, constitutes another support for adolescents’ reckless behaviors when coping with anxiogenic situations.

## ARE TEENS MORE VULNERABLE TO DRUG ABUSE THAN ADULTS?

Higher impulsivity is considered to promote drug first use, and eventually may lead to an increased vulnerability to develop drug addiction, defined as a loss of control over drug consumption and a compulsive pattern of drug use (Belin et al., 2008). Impulsivity is not easily defined (Evenden, 1999; Chamberlain and Sahakian, 2007), but a broad definition would include lack of attention, difficulty to suppress or control a behavioral response, pronounced novelty-seeking behavior, inability to anticipate consequences, difficulty to plan actions or reduced problem-solving strategies as key features. Because adolescents display more impulsive behaviors, the link between impulsivity and drug consumption has been extensively studied.

Converging studies using self-report questionnaire in teens demonstrated that impulsivity during adolescence was predictive of drug use and gambling (Romer et al., 2009), smoking initiation (O’Loughlin et al., 2009) and later alcohol abuse (Ernst et al., 2006; von Diemen et al., 2008). Reciprocally, impulsivity appeared to be exaggerated in adolescents with alcohol use disorders compared to healthy control (Soloff et al., 2000). Further, a study assessing genetic polymorphism has also demonstrated that a particular allele (A1) from the Taq1a polymorphism of the dopamine D2 receptor gene was positively correlated with alcohol and drug use (Esposito-Smythers et al., 2009). Concomitantly, impulsive carriers of the allele reported significantly more alcohol and drug-related problems than impulsive non-carriers. These findings highlight the interaction between vulnerability factors in the propensity to develop psychiatric troubles.

Cognitive impulsivity, defined as an inability to consider future outcomes, is a subdivision of impulsivity that takes into account emotional subjective representation of a delayed outcome. This concept is known as the discounting value of a reward (Rachlin, 1992). The use of the delay discounting, which offers to choose between immediate low rewards and future higher rewards, has contributed to better understand the neurobiological underpinnings of economic choice and decision-making. Adolescent tobacco smokers were found to be more impulsive than their non-smoker couterparts in a delay discounting task, and more prone to novelty seeking (Peters et al., 2011). Interestingly, the same group of adolescent smokers showed a marked decrease of striatal activation during a reward anticipation paradigm, which was positively correlated with smoking frequency. It is important to note that the increased impulsiveness reported in adolescent smokers might be a consequence, and not a predictor, of the addicted behavior. Studies comparing current and ex-smokers suggested that enhanced delay discounting curve concerns only current smoker (Bickel et al., 1999, 2008). However, other studies revealed that cognitive impulsivity could constitute a possible predictor of later substance use. Naíve adolescents, having a first cigarette smoking experience, were more impulsive in a delay discounting task (Reynolds and Fields, 2012). Nicotine intoxication is most likely not responsible for such results; it may rather reflect a personality trait shared by most of the adolescent smokers. Higher propensity to impulsive choices was also found to be predictive of the first ecstasy use in females (Schilt et al., 2009), and was also associated with binge drinking (Xiao et al., 2009).

It has been suggested that impulsivity represents a good index to predict the outcome of a smoking-cessation program: adolescents screened for higher impulsive trait significantly failed to maintain abstinence compared their non-impulsive counterparts (Krishnan-Sarin et al., 2007). Cognitive therapies targeting impulsivity, as reviewed elsewhere (Moeller et al., 2001), may constitute untapped opportunities for developing new approach to develop effective self-control in adolescents. This may contribute to prevent reckless behaviors occurring during this period of important morbidity.

## MODELING THE ADOLESCENT VULNERABILITY TO DRUG ABUSE

Brain development in juvenile rodents has been reported to display similar patterns resembling those of human beings, suggesting that the rodent model might be relevant to study the neurobiological underpinnings of teenage brain maturation (Spear, 2000). The juvenile period in rodents lasts from day 28 to day 42 after birth, but these limits, a bit restrictive, are usually extended to include a larger period from day 25 to day 55 (Tirelli et al., 2003). Neuroanatomical studies have described a massive synaptic pruning of dopamine receptors during adolescence in rodents (Andersen et al., 2000): D1 and D2 receptors density increased in the Nacc, the striatum and the PFC until the age of 40 days, and then progressively declined during early adulthood. Conversely, D3 receptors increased until 60 days (Stanwood et al., 1997). Another study revealed an increase of dopamine fibers in the medial PFC soon after weaning (Benes et al., 2000), that was in part controlled by the serotoninergic system: neonatal lesion of the raphe nucleus led to an increase of dopamine (DA) fibers sprouting from the ventral tegmental area (VTA) and the substantia nigra. Additionally, glutamatergic innervations from the PFC to the Nacc (Brenhouse et al., 2008) and to the amygdala (Cunningham et al., 2002) has been shown to follow a linear sprouting from weaning age to early adulthood. Dopaminergic modulation during adolescence appeared to be not entirely functional: the effects of D1 and D2 agonist on GABAergic interneurons in the PFC were weaker in adolescent, suggesting an uncompleted maturation of this modulatory system (Tseng and O’Donnell, 2007).

Behavioral studies comparing juvenile and adult rodents revealed that mice displayed a greater preference for a novel environment (Adriani et al., 1998), and enhanced impulsive responses compared to adults in a delay discounting task (Adriani and Laviola, 2003). Juvenile rodents also expressed a higher level of social interaction since social interactions were found to be more rewarding in juvenile than in adults rodents in a conditioned place preference (CPP) paradigm (Douglas et al., 2004). In line with this observation, a study reported that juvenile rats had lesser activation of dopamine signaling in the Nacc when facing non-social stimuli, but a more persistent response to social stimuli compared with adults (Robinson et al., 2011). This might reflect the importance of social interaction in juvenile animals.

In the elevated plus maze, adolescent rats spent a reduced period of time in the open arms, indicating a higher anxiety (Doremus et al., 2003; Estanislau and Morato, 2006; Lynn and Brown, 2010) although mice displayed a reversed profile (Macrì et al., 2002). Similar observations were reported using a contextual fear conditioning: adolescent rats froze significantly more than adults (Anagnostaras et al., 1999; Brasser and Spear, 2004; Esmoris-Arranz et al., 2008), but again adolescent mice froze less than adults (Pattwell et al., 2011).

With regards to the aversive effects of drugs, it has been shown that nicotine, ethanol, THC, amphetamine and cocaine induced less aversive effects in adolescent than in adult animals. In addition, conditioned taste aversion performed with a non-addictive substance (lithium chloride that induces abdominal pain after i.p. injections) is reduced in adolescent rats suggesting that insensitivity to aversive effects may be a generalized feature of adolescence (Philpot et al., 2003; Wilmouth and Spear, 2004; Schramm-Sapyta et al., 2006, 2007; Quinn et al., 2008; Drescher et al., 2011).

Meanwhile, several studies have reported increased reward sensitivity in juvenile animals. Nicotine and alcohol were found to be more rewarding in young rodents compared with adults (Philpot et al., 2003; Brielmaier et al., 2007; Kota et al., 2007; Torres et al., 2008; Spear and Varlinskaya, 2010). Similarly, increased sweetened condensed milk consumption (relative to body weight) was observed in adolescent rats compared with older ones. This behavioral observation was correlated with an increased c-fos expression in the Nacc core and the dorsal striatum (Friemel et al., 2010). Investigations assessing the effect of psychostimulants in adolescent rats using a CPP task remain a bit controversial, but a greater reward sensitivity in adolescents rats, particularly at lower doses, has been claimed in specific conditions (Badanich et al., 2006; Brenhouse et al., 2008; Zakharova et al., 2009).

## FACTORS INFLUENCING DRUG ABUSE IN ADOLESCENT RODENTS

Motor impulsivity refers to behavioral disinhibition and loss of impulse control, without necessary integration of emotional processing (Brunner and Hen, 1997). In animals, many behavioral tests have been shaped to assess this form of impulsivity, such as the five-choice serial reaction time task (5-CSRTT) and the differential reinforcement of low-rate (DRL). To our knowledge, the only study comparing impulsivity in non-treated normal adult and adolescent rats revealed that the latter were more impulsive in a DRL schedule (Andrzejewski et al., 2011). Prenatal exposure to nicotine has been shown to increase impulsivity in a 5-CSRTT during adolescence (Schneider et al., 2012), and chronic exposure to nicotine in adolescent rats produced long-lasting increase of motor impulsivity during adulthood (Counotte et al., 2009, 2011). In this study, nicotine chronic treatment was able to induce more impulsive behaviors on the 5-CSRTT when occurred during adolescence than during adulthood. This specific alteration, which did not affect cognitive impulsivity in a delay discounting task, has been correlated with a stronger nicotine-induced dopamine release in the PFC in adolescent rats. Similarly, impulsive adolescents, screened with the latency to approach a novel object, displayed an enhanced DA response to a cocaine challenge compared to non-impulsive adolescents or impulsive young adults (Stansfield and Kirstein, 2005).

However, prenatal treatment with nicotine, shown to alter motor impulsivity, failed to alter behavioral responses in a delay-discounting task (Schneider et al., 2012). While the influence between cognitive impulsivity and drug-seeking behaviors has been well established in humans, supplementary observations will be necessary to understand how it works in rodents. Diergaarde et al. (2008) have proposed that, at least in adult rats, motor impulsivity may be related to the initiation of drug seeking, while cognitive impulsivity may be associated with a decreased ability to suppress an acquired nicotine-seeking behavior and increased vulnerability to relapse. Ultimately, motor impulsivity, but not cognitive impulsivity might be more appropriate to assess drug-seeking vulnerability in juvenile rats.

Some basal differences of Hypothalamo-Pituitary-Adrenal (HPA) axis regulation may underlie an increased sensitivity to stressful stimuli in adolescent rodents. After an acute stress, adolescent rats displayed a higher adrenocorticotropic hormone (ACTH) and corticosterone release compared to adults (Romeo et al., 2006a,b). After a 30-min chronic restraint stress every day during 7 days, juvenile rats exhibited higher corticosterone levels immediately after the stressor, but corticosterone levels return to baseline values faster in adolescent than in adult rats (Romeo et al., 2006a). Male rats have been found to be more sensitive than females to the deleterious effects of maternal separation on PFC thickness (Spivey et al., 2009). Given the relations between stress and drug-seeking behaviors (Shaham et al., 2000; Koob and Le Moal, 2001), this increased sensitivity of the stress system may explain why some adolescents persist in drug abuse. A chronic cocaine treatment during adolescence increased several measures of anxiety when animals had become adults (Stansfield and Kirstein, 2005), which may further explain this persistence.

Compared to controls, rats stressed for 7 consecutive days during adolescence showed higher nicotine-induced enhancement of locomotor activity; this effect was not reported when stress occurred during adulthood (Cruz et al., 2008). Adolescent rats exposed to either a chronic restraint stress or a multiple-stress protocol showed higher locomotor response to cocaine challenge, and higher basal corticosterone level as well (Lepsch et al., 2005). Social stresses during adolescence increased behavioral sensitization to amphetamine (Mathews et al., 2008), but opposite effects were also reported (Kabbaj et al., 2002). Maternal separation was shown to increase impulsivity and reward-seeking behaviors (Colorado et al., 2006). Three hours of maternal separation between PND 0 and PND 14 increased the locomotor sensitization to cocaine, which was associated with an increase in D3R mRNA in the Nacc shell (Brake et al., 2004). Nevertheless, another study found no effect using a chronic social isolation on the locomotor response to psychostimulants either in adolescent or adult male rats (McCormick et al., 2005).

## THE JUVENILE RODENT MODEL: PROMISES AND PITFALLS

Most studies point out to an increased drug-seeking behavior in juvenile rodents, suggesting work hypotheses to explain why teens are at risk to lose control over drug intake. First, enhanced sensitivity to drug reward and two, lowered drug-induced aversive side effects provide a good rationale for studying juvenile rats vulnerability to drug abuse. However, no animal study has so far directly demonstrated an increased susceptibility to compulsive drug intake when first drug intoxication occurs during adolescence. Some methodological issues may also promote some misinterpretations, such as the lack of appropriate adult controls. As mentioned above, rats and mice appear to exhibit opposite anxiety profiles, with juvenile rats more anxious and juvenile mice less anxious than adults (Macrì et al., 2002; Lynn and Brown, 2010). Importantly, a few studies illustrated behavioral differences between early, mid and late adolescence (Tirelli et al., 2003; Wilkin et al., 2012), but most studies actually used juvenile rats of different ages that differed from one lab to the other. Further, the lack of consideration of social influence on drug consumption and related behavior may constitute another important confounding factor. Indeed, social interactions have been shown to highly influence risky behaviors and drug abuse. In particular, it has been reported that social interaction linked to a suboptimal cocaine dose could produce a CPP (Thiel et al., 2008). Meanwhile, the presence of counterparts decreased the aversive effect of ethanol in a conditioned taste aversion paradigm in male adolescent rats, but not in adults (Vetter-O’Hagen et al., 2009).

Ventral tegmental area dopaminergic neurons have been claimed to fire at a higher rate in adolescent rats, which is consistent with the hypothesis of adolescent vulnerability to drug abuse (McCutcheon et al., 2012). In line with this observation, a higher drug-induced dopamine release has been reported in adolescent rodents (Laviola et al., 2001; Walker and Kuhn, 2008). However, behavioral response to drugs does not fit with this conclusion. In particular, subchronic treatment with psychostimulants failed to induce an increased locomotor sensitization in adolescent rats (Frantz et al., 2007). Of particular importance, Frantz et al. (2007) reported similar dopamine release in the Nacc between adolescents and adults rats treated with psychostimulants. Conversely, one study reported a locomotor sensitization to cocaine in juvenile mice and not in adults (Camarini et al., 2008); however, a cocaine challenge performed 10 days after this experiment showed a lower dopamine release in the Nacc of juvenile mice, despite a faster onset peak. Further studies will be necessary to determine the relation between DA release and locomotor sensitization to psychostimulants in adolescent rats.

Although stress and impulsivity have been shown separately to promote drug use, a few studies established cross-regulations between both. Intracerebroventricular injections of corticotropin-releasing factor (CRF) did not increase impulsivity in the 5-CSRTT, but increased accuracy responding (Ohmura et al., 2009). A chronic treatment with corticosterone during adolescence failed to affect premature responses in this task, and even decreased the number of impulsive behaviors in a Stop signal task (Torregrossa et al., 2012). More studies are needed to fully understand this interaction, which is considered as a key element exaggerating the emergence of psychiatric disorders in human (Fox et al., 2010; Somer et al., 2012; Hamilton et al., 2013).

Another source of controversy is the conjecture according to which the juvenile rodents would exhibit reduced self-control and increased attraction to cues predicting reward (Ernst et al., 2009; Burton et al., 2011). In opposition with this statement, juvenile rats were shown to display a lower cue-induced reinstatement of cocaine intake (Anker and Carroll, 2010). Further contrasting with the above mentioned conjecture, juvenile mice (26–27 days) were shown to exhibit enhanced flexibility compared to adults in an odor-cue based procedure (Johnson and Wilbrecht, 2011). Given the immaturity of the PFC in juvenile rats, as well as the key role of this structure in cognitive flexibility (Baxter et al., 2000; Schoenbaum et al., 2006; Gruber et al., 2010), this result might appear counterintuitive. Nonetheless, an enhanced flexibility of adolescents might help to promote a switch between a large number of options, such as quitting drug intake in favor of a less detrimental behavior. It therefore tends to alleviate the omnipresence of vulnerability elements in juvenile rodents, since cognitive flexibility is mandatory to acquire a behavioral repertoire necessary for survival and autonomy.

It is important to acknowledge that only a minority of youngsters experiencing recreational drugs will later develop clinical symptoms of drug addiction and dependence, although the contribution of fundamental research using animal models remains quite limited to support this assertion. A current consensus suggests that interindividual variations in brain maturation might explain excessive behavioral outputs. Of particular interest, recent evidence demonstrated that first, individuals with pronounced impulsive traits displayed a thinner cortex (Shaw et al., 2011) and second, the activation of the mesolimbic neurocircuitry of adolescents trained to gamble in a monetary incentive task correlated positively with their psychosocial and behavioral difficulties (Bjork et al., 2011). The authors of this study elegantly acknowledge that correlation most likely does not imply causality but, nonetheless, these observations suggest that increased engagement in problematic behaviors may partly result from mesolimbic sensitivity to reward-predictive cues. And they conclude that increased mesolimbic sensitivity may represent a trait that, in line with the general immaturity of the adolescent brain, could partly explain behavior-related injury or death in “at-risk” adolescents (Bjork et al., 2011).

Some external factors, like sociodemographic status or familial environment, have also been considered to play a role in this variability. Adverse events in childhood were shown to be predictive of later alcohol dependence (Pilowsky et al., 2009). Converging evidence has established the negative influence of parental misconducts (including substance use disorders) on children propensity to develop similar disorders (Verdejo-Garcia et al., 2008). Gene polymorphisms among adolescents with alcohol-related disorders have been proposed to explain interindividual differences in attentional bias toward alcohol (Pieters et al., 2011), or in stress responsivity to drugs (Kreek et al., 2005). Although genetic factors have been thought to explain between 30 and 60% of addictive disorders (Kreek et al., 2005), gene influence mainly depends on interaction with environmental factors. In particular, a gene polymorphism was shown to be closely related to alcoholism in adults, and also in a subpopulation of adolescents that were exposed to high psychosocial stress during childhood (Clarke et al., 2011). A similar correlation has been found with a specific genotype of the serotonin transporter (Kaufman et al., 2007). In adolescents diagnosed for anxiety disorders, depression, or in healthy controls, amygdala pattern of activation in response to emotional faces was dependent of the pathology diagnosed (Beesdo et al., 2009).

## CONCLUSION

Risk taking and sensation seeking have long been considered hallmarks of typical adolescent behavior and, meanwhile, have been thought to represent vulnerability factors for developing substance abuse disorders. Strikingly, despite a large number of preclinical investigations delineating the brain circuitries underpinning enhanced impulsiveness and increased emotional reactivity constitutive of an extended behavioral repertoire, very few studies support a specific vulnerability of juvenile rodents to lose control over drugs of abuse. A provocative statement would argue that science should better see the adult world with adolescent eyes, rather than seeing the adolescent world using an adult watch. Indeed, juvenile behaviors present adaptive benefits to acquire appropriate skills for survival in absence of parental protection. Meanwhile, it is true that these externalizing behaviors make adolescents, or at least a subset of teens, more vulnerable to reckless conducts and potential injuries. Objectively, the adolescent brain is prewired for sensation seeking and risk taking which, in line with the heightened motivation for reward, often leads to careless behaviors. The development of self-regulatory competence is a normative process (that depends on both brain maturation and social experiences) at the end of which young adults have acquired the aptitude to better regulate their emotions and impulsiveness.

A major aim for future researches consists in finding endophenotypes and vulnerability markers of substance use disorders and drug abuse. It has been recently demonstrated that people suffering from substance abuse disorders shared with their non-addict siblings similar behavioral traits, including high impulsivity and sensation-seeking (Ersche et al., 2010). This study also revealed that abnormal prefrontal and striatal connectivity might underpin risks of drug addiction (Ersche et al., 2012). In complement, converging evidence have revealed that interindividual differences arise from heterogeneity in the PFC function (George and Koob, 2010). Therefore, deeper investigations assessing PFC interindividual adaptations during adolescence are required to understand how only specific developmental trajectories can lead to drug addiction. In particular, understanding whether (and if true, how) deficient brain maturation processes might be responsible for sustained reward seeking and poor decision-making (meaning persistence in risk taking despite adverse consequences) is of the highest importance to better protect “at-risk” young adults. A current consensus already acknowledges that the developing adolescent brain is fragile and vulnerable to neurobiological insults concomitant to drug abuse, in particular those related to alcohol intoxication (Crews et al., 2004). But, further preclinical and clinical studies focusing on the adolescent PFC are required to better understand how genes, environment, stress and individual temperament interact together to shape the neurobiological mechanisms underpinning the vulnerability to lose control over reward seeking, and potentially excessive drug taking, during the transition from the adolescent world to the adult universe.

## Conflict of Interest Statement

The authors declare that the research was conducted in the absence of any commercial or financial relationships that could be construed as a potential conflict of interest.
